# Employees’ perceptions of patient safety culture in Norwegian nursing homes and home care services

**DOI:** 10.1186/s12913-019-4456-8

**Published:** 2019-08-29

**Authors:** Eline Ree, Siri Wiig

**Affiliations:** SHARE – Centre for Resilience in Healthcare, Faculty of Health Sciences, University of Stavanger, N-4036 Stavanger, Norway

**Keywords:** Patient safety culture, Home care services, Nursing homes, Incident reporting, Patient safety, Teamwork, Staffing

## Abstract

**Background:**

Most health care services are provided in the primary health care sector, and an increasing number of elderly is in need of these services. Nonetheless, the research on patient safety culture in home care services and nursing homes remains scarce. This study describes staff perceptions of patient safety culture in Norwegian home care services and nursing homes, and assesses how various patient safety culture dimensions contribute to explaining overall perceptions of patient safety.

**Methods:**

Cross-sectional surveys were conducted among healthcare professionals in Norwegian home care services (*N* = 139) and nursing homes (*N* = 165) in 2018, response rates being 67.5% and 65%, respectively. A Norwegian version of the international recognized Nursing Home Survey on Patient Safety Culture was used. Descriptive statistics and t-tests were used to explore staff perceptions of patient safety culture. We used multiple regression analyses to explore the degree to which patient safety culture dimensions could explain overall perceptions of patient safety.

**Results:**

The number of patient safety dimensions having an average score of more than 60% positive responses was seven out of 10 in nursing homes, and nine out of 10 in home care. Staffing had the lowest scores in both health care services. Home care services scored significantly higher than nursing homes on teamwork (eta squared = .053), while nursing homes scored somewhat higher on handover (eta squared = .027). In home care, total explained variance of overall perceptions of patient safety was 45%, with teamwork, staffing, and handoffs as significant predictors. The explained variance in nursing homes was 42.7%, with staffing and communication openness as significant predictors.

**Conclusions:**

There are differences in perceptions of patient safety culture between nursing homes and home care services. Staffing is important for patient safety perceptions in both health care services. In home care, teamwork seems to be a significant contributing factor to patient safety, and building sound teams with mutual trust and collaboration should therefore be an essential part of managers’ work with patient safety. In nursing homes, the main focus when building a good patient safety culture should be on open communication, ensuring that staff’s ideas and suggestions are valued.

## Background

In this study, we describe staff perceptions of patient safety culture in Norwegian home care services and nursing homes, and explore how different dimensions of patient safety culture can predict overall perceptions of patient safety.

Fifteen years after the well-known report of the Institute of Medicine (IOM), which found that medical errors caused between 44,000 to 98,000 deaths in the U.S. each year [1], a new IOM report concludes “the pace and scale of improvement has been disappointingly slow and limited” [2]. The latest report emphasizes the need to promote patient safety culture in all health care settings, not just in inpatient settings such as hospitals, where most of the research has been conducted. This emphasis is also evident in Norwegian governmental policies and guidelines. In 2014 the Norwegian Ministry of Health and Care services implemented a national patient safety program emphasizing the need to improve patient safety culture in the health care services, including nursing homes and home care services [3]. A recent national action plan for patient safety and quality improvement states that there are still too many patient injuries and adverse events in the health care services [4]. The report emphasizes management and safety culture as key target areas, and suggests the development of a culture that facilitates openness and learning. Several national white papers address the challenges faced by a continuously aging population, leading to greater pressure on the municipalities as more people than ever receive their health care in primary care settings [5, 6]. The establishment of sound structures and cultures for safety is key in these programs and plans.

A recently published systematic review concludes that addressing and understanding patient safety culture is the most important first step in improving patient safety in primary care [7]. A patient safety culture is commonly defined as “the product of individual and group values, attitudes, perceptions, competencies, and patterns of behavior that determine the commitment to, and the style and proficiency of, an organization’s health and safety management” [8]. Several studies stress the importance of patient safety culture for patient safety processes and outcomes [9]. More specifically, research has shown that a sound patient safety culture is associated with fewer adverse events [10, 11] and more positive patient experiences [12]. A systematic review by Braithwaite et al. [9] found that a positive workplace culture was related to several desirable patient outcomes, such as fewer falls and infections, reduced rates of mortality, and increased patient satisfaction. These findings were consistent across countries, settings, and studies, including aged care facilities.

Despite increased attention to quality and patient safety issues in primary care, there is a large knowledge gap, especially in the home care setting. In addition, most studies are conducted in the U.S. [13]. Some studies of patient safety culture in Norwegian nursing homes have been conducted recently [14–16]. To our knowledge, only one study has explored patient safety culture in Norwegian home care settings [17]. Thus, there is a need for more research on patient safety culture in these settings in order to identify possible areas for improvement and to develop more targeted interventions in these contextual settings.

As far as we know, no studies have compared perceptions of patient safety culture in the nursing home and home care settings. Despite several similarities, nursing homes and home care services have significant differences in terms of structure (institutional vs. home care) and in the burden of disease or illness among the patients/users of the services. It is therefore possible to assume that there will be different patterns in how employees in the two organizations score on patient safety culture dimensions, and which dimensions best predict an overall perception of patient safety. The aims of this study were therefore to explore:

1. the scores on patient safety culture dimensions in Norwegian nursing homes and home care services
2. differences between nursing homes and home care services on patient safety culture dimensions
3. the degree to which the different dimensions of a patient safety culture predict employees’ overall perceptions of patient safety in nursing homes and home care services

## Methods

A cross-sectional survey design was conducted in this study.

### Setting and sample

The sample was selected purposively based on the units’ participation in an ongoing large intervention project [18], and consisted of a total of 304 health care personnel, most of whom were females. Of these, 165 were employed in nursing homes, and 139 in home care services. The distribution of age, gender and working experience was similar in the two organizations (Table 1), and is relatively similar to distributions in other Norwegian nursing homes [15].

**Table 1:** Relevant background variables of the respondents in the home care services (*n* = 139) and the nursing homes (*n* = 165)

Background variables	Home care *n* (%)	Nursing homes *n* (%)
Age		
20–29 years	14 (10.1)	20 (12.1)
30–39 years	33 (23.7)	39 (23.6)
40–49 years	40 (28.8)	26 (15.8)
50–59 years	38 (27.3)	50 (30.3)
60 + years	14 (10.1)	30 18.2)
Staff position		
Managers including leaders at first-line level	9 (6.5)	11 (6.7)
Healthcare workers with a minimum of bachelor degree	59 (42.4)	67 (40.6)
Healthcare workers, upper secondary school	56 (40.3)	79 (47.9)
Assistants	12 (8.6)	3 (1.8)
Others	3 (2.1)	5 (3.0)
Number of years in current workplace		
< 1 year	6 (4.3)	20 (12.1)
1–5 years	38 (27.3)	41 (24.8)
6–10 years	35 (25.2)	27 (16.4)
11–15 years	19 (13.7)	24 (14.5)
16–20 years	29 (20.9)	20 (12.1)
> 21 years	12 (8.6)	33 (20.0)

### Data collection

Four home care units and four nursing home units from five municipalities in southwestern Norway participated in the study. The sample was strategically selected in order to represent variations in the size of municipality and units, as well as their location (Table 2). Since the study is part of the SAFE-LEAD primary care project, the selection criteria in the project implied inclusion of units from different municipalities and contextual settings in Norway (see study protocol for further details in Wiig et al. [18]). Recruitment was conducted by co-researchers from the Development Centres of Nursing Home and Home Care Services in the project [18], by contacting the managers in all participating units. The surveys were distributed in 2018 by email, using SurveyXact, and the response rates were 65% on the nursing home survey and 67.5% on the home care survey. To be included, participants had to be employed in a minimum of 30% position, and speak Norwegian.

**Table 2:** Survey administration and characteristics according to municipality size and response rate

	Municipality size (ca N of inhabitants)	Surveys completed *n* (response rate)
HCS 1	15–20,000	65 (86.6%)
HCS 2	70–75,000	22 (56%)
HCS 3	< 5000	26 (56%)
HCS 4	5–10,000	25 (53.2%)
NH 1	130–135,000	90 (73.2%)
NH 2	70–75,000	23 (69.7%)
NH 3	< 5000	26 (45%)
NH 4	5000–10,000	26 (65%)

### Questionnaire

To measure patient safety culture, we used the Norwegian version of the Nursing Home Survey on Patient Safety Culture (NHSOPSC). The questionnaire is originally developed and validated by the Agency for Healthcare Research and Quality [19]. The instrument is validated in Norwegian nursing homes by Cappelen et al. [14]. In this study, some items were slightly modified to fit the home care setting in the questionnaire sent to employees in the home care services, by replacing the word ‘nursing home’ with ‘unit’ or ‘home care service’, and by replacing ‘patient’ with ‘user’. The instrument consists of 41 items rated on 5-point Likert scales, measuring the perceptions of health personnel on different dimensions of patient safety culture. Previous studies have documented acceptable fit in a Norwegian setting for a 10-factor model of the scale [14, 15]. These factors are as follows: ‘teamwork, staffing, compliance with procedures, training and skills, nonpunitive responses to mistakes, handoffs, feedback and communication about incidents, communication and openness, supervisor expectations and actions promoting patient safety, and organizational learning’. Questions regarding frequency of event reporting are included in the NHSOPSC, measured by the following items: ‘how often are near misses events reported – i.e., events that is caught and corrected before affecting the patient?’, and ‘how often are potential harmful events reported – i.e., events that could harm the patient, but do not?’. The items were rated on a five-point Likert scale from 1 = never to 5 = always.

The questionnaire also included a single item on overall perceptions of patient safety, which was used as an outcome variable. Overall perception of patient safety was measured with the item ‘Please give this nursing home/unit an overall rating on patient safety’, rated on a five-point Likert scale from 1 = ‘very poor’ to 5 = ‘very good’.

### Statistical analyses

For all the statistical analyses we used IBM SPSS Statistics version 25. The respondents had to answer each question before being able to advance to the next, so we have no missing values in the dataset.

Descriptive frequency analyses were used to explore staff perceptions of patient safety culture. On items that were positively worded, responses on 4 and 5 (‘agree/strongly agree’ or ‘most of the time/always’) on the 5-point Likert scale indicated positive responses, while 1 and 2 (‘strongly disagree/disagree’ or ‘never/rarely’) indicated positive responses on negatively worded items. The total percentages of positive scores per dimension were calculated by adding the percent of positive scores on all items divided by the number of items in each dimension [15, 19]. Having an average percentage of 60% positive responses per patient safety culture dimension was considered a good score, as this is shown to indicate a lower risk if adverse events [20]. Independent t-tests were conducted to explore whether the two organizations differed significantly on the patient safety culture dimensions, overall perceptions of patient safety, and on incident reporting. The effect sizes of the mean differences were calculated using Eta-squared.

Multiple regression analyses with overall perceptions of patient safety as outcome was conducted, with eight of the patient safety culture dimensions as predictors in each model. The two dimensions ‘non-punitive responses to mistakes’ and ‘management support and organizational learning’ were not included in the analyses due to multicollinearity and suppression effects [21]. To assess whether the associations differed between nursing homes and home care, the analyses were conducted independently for the two organizations, in addition to exploring the effects in the total sample.

## Results

### Staff perceptions of patient safety culture

**Fig. 1:**
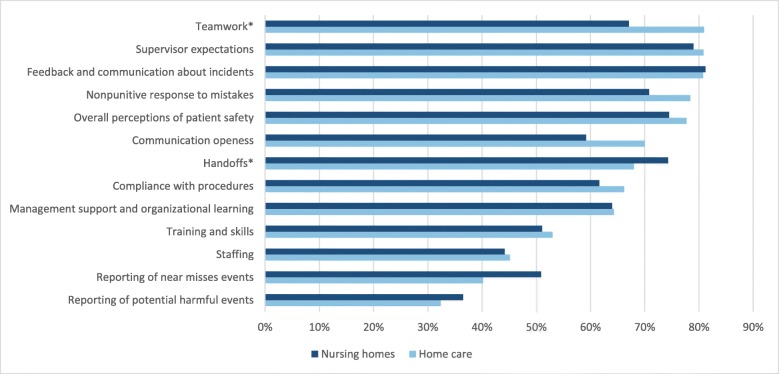
Average percentage positive responses per patient safety culture dimension, frequency of reporting of events, and overall perceptions of patient safety in home care and nursing homes. *differences significant at *p* < .05

Seven out of 10 patient safety dimensions in nursing homes, and nine out of the 10 dimensions in home care services had an average score of more than 60% positive responses (Fig. 1). The home care services had higher scores than nursing homes on all variables, except on reporting of potential harmful events and near misses events, and handoffs. The average percentage of positive scores was the same in nursing homes and home care regarding ‘management support and organizational learning’ and feedback and communication about incidents. In both nursing homes and home care, the lowest scores were on frequency of incident reporting. Among the 10 patient safety culture dimensions, the lowest scores were on staffing.

Results from t-tests showed that the only significant differences between nursing homes and home care services were found on the ‘teamwork’ and ‘handover’ dimensions. The size of the mean differences (MD = −.31, 95% *CI:* 0.46 – − 0.16) was moderate (eta squared = .053) for the difference between nursing homes (M = 3.83, SD = 0.73) and home care services (M = 4.13, SD = 0.58; t (302) = − 4.08, *p* = .000, two-tailed) on teamwork. For handover, the size of the mean differences (MD = .19, 95% CI: 0.06–0.32) was small (eta squared = .027) between nursing homes (*M* = 3.97, SD = 0.65) and home care services (*M* = 3.78, *SD* = 0.50; *t* (302) = 2.90, *p* = .004, two-tailed).

### Multiple regression analyses of overall perceptions of patient safety

In the multiple regression analyses of overall perceptions of patient safety, with the patient safety culture dimensions as predictors, teamwork, staffing, communication openness, and supervisor expectations were significant predictors in the total model including both nursing homes and home care (R2 = .426, *p* < .001) (Table 3). The total explained variance was higher in the home care model (R2 = .450, *p* < .001) than in the nursing home model (R2 = .427, *p* < .001). Teamwork was the strongest predictor for overall perceptions of patient safety in home care services, followed by staffing in handoffs. In nursing homes, the only significant predictors were communication openness, and staffing.

**Table 3:** Multiple regression analyses of overall perceptions of patient safety in nursing homes, home care services, and in total for the two samples

Variables	Overall perceptions of patient safety								
	Nursing homes (*n* = 165)			Home care (*n* = 139)			Total (*n* = 304)		
	B	SE	*β*	B	SE	*β*	B	SE	*β*
Teamwork	.079	.105	.070	.315	.101	.254*	.156	.069	.137*
Staffing	.217	.105	.158*	.252	.097	.215*	.244	.071	.190*
Compliance with procedures	.031	.091	.024	.070	.096	.055	.037	.064	.029
Training and skills	.071	.106	.062	.005	.112	.004	.047	.075	.040
Handoffs	.050	.126	.039	.256	.129	.178*	.145	.086	.110
Feedback and communication about incidents	.223	.145	.166	.080	.124	.058	.135	.094	.099
Communication openness	.203	.101	.189*	.110	.110	.097	.161	.072	.147*
Supervisor expectations	.141	.083	.137	.138	.106	.124	.142	.062	.134*
R^2^			.427**			.450**			.426**

*p < .05. **p < .001

## Discussion

This study shows that patient safety culture explained a high percentage of the variance in overall perceptions of patient safety in both home care and nursing homes. However, the results revealed some notable differences between nursing homes and home care services in terms of patient safety culture perceptions, and which dimensions are most important for health personnel’s overall perceptions of patient safety. Teamwork was among the dimensions with the highest percentage of positive scores in the home care services, and home care services scored significantly higher than nursing homes on this dimension. Nursing homes scored significantly higher than home care on handover, although the mean difference was small.

As far as we know, no studies have compared home care and nursing home settings in terms of patient safety culture perceptions. A previous study in Norwegian nursing homes reported positive responses of more than 60% on eight of the 10 patient safety culture dimensions [15], compared to seven dimensions in the nursing homes in this study. In the study by Cappelen et al. [15] however, the percentages of positive responses were higher on all dimensions than in the current study, except on staffing where they were identical, and quite low (44%). Staffing was also reported as low in a study of health personnel in Turkish primary health care [22]. Furthermore, in line with the Turkish study, the frequency of adverse event reporting represented the dimension with the lowest percentage of positive scores.

Other studies have linked poor staffing to perceptions of poor patient safety and quality of care [23, 24]. Furthermore, poor staffing seems to be related to several patient safety outcomes such as increased patient mortality [25–27], and increased reporting of poor/failing patient safety [23]. Other studies, including those of doctors have shown that doctors are more likely to report incidents to their colleagues than to their superiors [28]. Although doctors were not included in our sample, our results might indicate that a high score on teamwork is associated with less reporting of adverse events. In this study, participants in the home care services reported fewer adverse events than participants in nursing homes, but they scored significantly higher on teamwork. In a good team with mutual trust among colleagues, events might be discussed and ‘solved’ within the team, and they might feel the obligation not to report their colleagues’ mistakes. However, the opposite may also be true, as identified in Edmondson’s [29] work on psychological safety. Edmondson found that in teams characterized by psychological safety, where staff dare to speak up about their ideas and worries, people report more than they do in teams with less psychological safety. This finding is supported by other studies [30–32], which indicate there is a need to explore the relationship between teamwork, error reporting, and learning from adverse events in nursing homes and home care. In addition, the role of communication and work culture for creating psychological safety should be investigated in the primary care settings.

Despite representing the lowest percentage of positive scores, incident reporting was still higher in this Norwegian study than in international studies [22, 33]. Furthermore, participants in this study scored considerably higher on the dimension ‘non-punitive response to mistakes’, which is consistent with other Norwegian studies [15, 34, 35]. International studies report low scores on this dimension, both in the primary care [21, 36, 37], and in hospitals and outpatient settings [21, 33, 38]. This can probably be explained by Norway’s unique health care settings, which are uniquely open and non-hierarchical in structure [39]. In the U.S. for example, lawsuits and individual performance schemes are much more common [40], than in Norway.

### Strengths and limitations

To our knowledge, this is the first study comparing patient safety perceptions in Norwegian nursing homes and home care services, assessing how various dimensions of the patient safety culture can explain overall perceptions of patient safety. Yet, the cross-sectional design of the study and the small samples limit the generalizability of the findings. The variability between the participating units regarding locations and municipality size as well as the heterogeneity in locations (urban/rural) is, however, representative for units across Norway. Future studies should explore perceptions of patient safety in these settings in a longitudinal design, including larger samples. Furthermore, future studies should investigate how the patient safety culture dimensions are linked to measurements of real patient safety, and whether patient safety culture interventions affect patient safety outcomes.

Although the relatively low response rate might have limited the validity of the results, it is still higher than or similar to other Norwegian studies in similar settings [15–17], indicating a general difficulty of achieving high response rates in these settings. The response rates were lower in some of the units. However, there were no statistically significant differences between the units with a low response rate and the other units on the patient safety culture dimensions. Moreover, the distributions were similar with regards to background variables, reducing the risk of response bias. Nevertheless, a larger sample would allow for sub-group analyses among different wards and departments within units, as well as between occupations, levels of education and working experience. A recent study found differences between doctors’ and nurses’ ratings of patient safety culture [41]. Furthermore, studies of Norwegian hospitals revealed considerable differences between wards and departments at the same hospital [35, 42], suggesting that measuring patient safety culture as a basis for improvement efforts should be conducted at all levels in an organization, as close to patient care as possible [42].

## Conclusions

Measuring patient safety culture in health care organizations can be an efficient way to map cultural challenges of importance for patient safety, and should be considered by managers when planning and implementing improvement efforts. This study has shown that Norwegian nursing homes and home care services have different challenges from what is often reported in international studies, and that there are differences across the two health care services. Training and skills, staffing, and reporting of incidents should be targeted in improvement efforts in both home care services and nursing homes. In the latter, there is also room for improvement in communication openness, as this dimension was predictive of overall perceptions of patient safety, yet health personnel in nursing home scored low on this dimension.

## Data Availability

The datasets analyzed in the present study and the Norwegian version of the questionnaire are available on request from the corresponding author.

## Abbreviations

IOM: Institute of Medicine
NHSOPSC: Nursing Home Survey on Patient Safety
